# Comparing Two Types of Robotic Single-Site Myomectomy Using Propensity Score Matching: Coaxial with da Vinci Xi vs. da Vinci SP System

**DOI:** 10.3390/jcm14145106

**Published:** 2025-07-18

**Authors:** Nara Lee, Su Hyeon Choi, Mi-La Kim, Sa Ra Lee, Seok Ju Seong

**Affiliations:** 1 Department of Obstetrics and Gynecology, CHA Gangnam Medical Center, CHA University, Seoul 06135, Republic of Korea; naradd@chamc.co.kr (N.L.); k345@chamc.co.kr (S.H.C.); mila76@chamc.co.kr (M.-L.K.); 2 Division of Urogynecology and Reproductive Endocrinology, Department of Obstetrics and Gynecology, Asan Medical Center, University of Ulsan, Seoul 05505, Republic of Korea

**Keywords:** da Vinci SP robot system, robot-assisted operations, fibroid surgery utilizing robotic technology, robotic single-site myomectomy, uterine myomectomy, uterine leiomyomas, propensity score matching

## Abstract

**Background**: This study was designed to evaluate and contrast the surgical outcomes between coaxial robotic single-site myomectomy (RSSM) performed using the da Vinci Xi system and da Vinci SP system. **Methods**: A retrospective review was conducted on 81 women who underwent coaxial RSSM and 108 women who underwent myomectomy with the da Vinci SP system between October 2020 and January 2024. Propensity score matching was performed based on myoma count, the dominant myoma’s maximum diameter, and the myoma type according to the International Federation of Gynecology and Obstetrics (FIGO) classification. Patient characteristics and surgical outcomes were evaluated and compared between the two groups. **Results**: Compared to the SP group, the coaxial RSSM group showed significantly lower estimated blood loss (102.33 ± 61.01 vs. 203.98 ± 163.15 mL, *p* < 0.001), shorter operative time (91.22 ± 18.25 vs. 148.69 ± 45.62 min, *p* < 0.001), and smaller hemoglobin decrement (1.69 ± 0.93 vs. 2.85 ± 1.30, *p* < 0.001). However, hospital stay was shorter in the SP group than in the coaxial group (2.06 ± 0.24 vs. 4.07 ± 0.76 days, *p* < 0.001). There were no statistically significant differences in postoperative complications, including ileus, fever, or wound dehiscence. Additional comparisons using cases performed by four different surgeons yielded results consistent with the one-to-one surgeon comparison. **Conclusions**: Coaxial RSSM was associated with a shorter operative time and lower blood loss compared to SP myomectomy. A prospective study is warranted to validate and further compare the surgical outcomes of the two techniques.

## 1. Introduction

Robotic myomectomy is a less invasive approach for removing uterine fibroids, with the da Vinci robot system (Intuitive Surgical., Inc., Sunnyvale, CA, USA) being widely adopted for this procedure [1,2,3,4,5]. The system offers several advantages, including a three-dimensional view of the surgical field, minimizing the surgeon’s hand tremors, and providing improved control of suturing [6]. The terminology used to describe minimally invasive robotic approaches—particularly “single-site” and “single-port”—is often used interchangeably in the literature, although their definitions and applications can vary across studies [6,7,8]. For clarity, in this study, robotic single-site myomectomy (RSSM) refers to procedures performed using the da Vinci Xi system with curved semi-rigid instruments inserted through a single umbilical incision [9]. Single-port robotic surgery refers to procedures using the da Vinci SP system, in which the camera and three articulated instruments are introduced through a single multichannel port. Coaxial RSSM, as defined in this study, is a modified RSSM technique using standard 8 mm rigid robotic instruments inserted coaxially through separate cannulas via a single incision to improve traction and suturing [10]. Single-site robot surgery is particularly noted for its superior cosmetic outcomes compared to multiport robot surgery, because it requires only one transumbilical entry point. Robotic single-site myomectomy (RSSM) has gained recognition as a viable option, demonstrating effectiveness and reliability comparable to single-port laparoscopic myomectomy and conventional robotic myomectomy [3,11]. However, RSSM has a notable drawback in terms of traction power during myoma retrieval [10]. The use of semi-rigid instruments and the lack of adequate counter-traction tools pose significant challenges in performing myomectomy with robotic single-site systems. Although a 5 mm laparoscopic tenaculum forceps can be inserted via an auxiliary port to facilitate counter-traction during myoma retrieval, the single-site opening leads to increased instrument collision and limited movement, which hinders effective manipulation.

To address the limitations of RSSM, the coaxial RSSM technique was developed [10,12]. Coaxial RSSM involves attaching conventional robotic cannulas and devices at a single entry point through different techniques, allowing the use of an 8 mm robust instrument for myoma retrieval and suturing. In our earlier study, we compared coaxial RSSM to RSSM, highlighting the surgical outcomes. Compared to conventional RSSM, coaxial RSSM required less operative time (101.0 vs. 146.1 min, *p* = 0.008) and lower estimated blood loss (75.0 mL vs. 210.5 mL, *p* = 0.001).

The da Vinci SP system (Intuitive Surgical) is the latest robotic platform, receiving FDA approval for urologic surgeries in 2018 [13,14]. Due to its relatively recent introduction, various challenges are likely to emerge as more institutions adopt and utilize this system. The SP system instruments have two joints, with the wrist joint providing 7 degrees of freedom, and the SP camera offering a 73-degree field of view. The SP system is useful for various gynecologic surgeries and may be advantageous in overcoming the limitations of conventional single-site robotic systems. However, the camera and up to three instrument devices enter the working space through a single port, and the field of view and working area in the SP system are more restricted compared to a multiport system. This can make it challenging for the surgeon to achieve an optimal view of the target structure during surgery. The da Vinci SP system is also being utilized for myomectomy procedures, but there is limited research on its surgical outcomes [15].

This study was conducted to evaluate and contrast the operative results of coaxial RSSM and procedures performed using the da Vinci SP.

## 2. Materials and Methods

### 2.1. Study Participants

This retrospective study, conducted from October 2020 to 2024, collected data from CHA Gangnam Medical Center, CHA University, and Asan Medical Center, Ulsan College of Medicine. Ethical approval for this study was obtained from the Institutional Review Board (IRB) of CHA Gangnam Medical Center (IRB No. 2024-01-022) and the IRB of Asan Medical Center (IRB No. 2025-0211). The study included patients with single or multiple myomas requiring treatment with either coaxial RSSM at CHA Gangnam Medical Center or da Vinci SP at Asan Medical Center. The criteria for single-site robotic surgery were based on the surgeon’s judgment, ensuring that the number of myomas did not exceed five and the largest myoma did not exceed 10 cm.

In total, 81 patients received coaxial RSSM, while 108 underwent surgery with da Vinci SP. To minimize surgeon-dependent variability, a comparative analysis was first performed between a single surgeon from CHA Gangnam Hospital and a single surgeon from Asan Medical Center (matched surgeon comparison). To further validate the findings, an additional analysis was conducted comparing the outcomes of four surgeons at CHA Gangnam Hospital with those of a single surgeon at Asan Medical Center (multiple-surgeon vs. single-surgeon comparison).

Of the 81 coaxial RSSM cases, 45 performed by a single surgeon were included in the one-to-one surgeon analysis to allow for direct inter-surgeon comparison. The full cohort was used in the multiple-surgeon vs. single-surgeon (four-to-one) analysis.

### 2.2. Variables

The database review collected information on various aspects including chronological age, body mass index (BMI), parity, prior history of abdominal operations, presence of peritoneal adhesions, and any simultaneous procedures. It also gathered details about the characteristics of the myomas, the duration of the surgery, preoperative and postoperative hemoglobin (Hb) levels, the need for transfusion, length of hospital stay, and any postoperative complications.

The myoma-related variables included the overall count of myomas excised, the diameter of the largest one (in centimeters), combined weight (in grams), and anatomical site of the dominant myoma, which was classified as anterior, posterior, fundal–anterior, fundal–posterior, fundal. Furthermore, the types of myomas were categorized according to the FIGO subclassification (type 0–8) and grouped as submucosal (type 0–2), intramural (type 3–5), subserosal (type 6–8). Patients who received transfusions during surgery or hospitalization were excluded. Preoperative Hb levels were measured within four weeks prior to the operation, and again on postoperative day one. The drop in hemoglobin (g/dL) was calculated by subtracting postoperative from preoperative hemoglobin. Operative time was measured from the initial skin incision to the final skin closure. Wound dehiscence was monitored throughout the first month following surgery.

### 2.3. Surgical Procedure

All patients were administered general anesthesia, including endotracheal intubation to maintain their airway during the procedure, and were placed in the dorsal lithotomy position, which offers optimal access to the pelvic area. After anesthesia and positioning, a Foley catheter was inserted into each patient to ensure continuous urinary drainage throughout the surgery. Additionally, to achieve precise control and positioning of the uterus, a uterine RUMI manipulator was introduced under aseptic conditions to minimize the risk of infection.

For coaxial RSSM, an approximately 2.5 to 3.0 cm incision was made at the umbilical site to insert a glove port (Nelis, Seoul, Republic of Korea). In contrast, for the SP-RSSM, transumbilical skin incision, measuring about 27 mm to 30 mm, was performed. The incision was then extended down to the fascial layer using the open Hassel technique, a widely used method for ensuring safe and controlled access during laparoscopic or robotic surgeries. After achieving proper access through the fascial layer, a specialized multichannel SP designed for the da Vinci^®^ SP robotic system was inserted. The specific port used for these procedures was either the Uni-port, manufactured by Dalim, Seoul, Republic of Korea, or the Gloveport, made by Nelis.

#### 2.3.1. Coaxial RSSM

**Figure 1:**
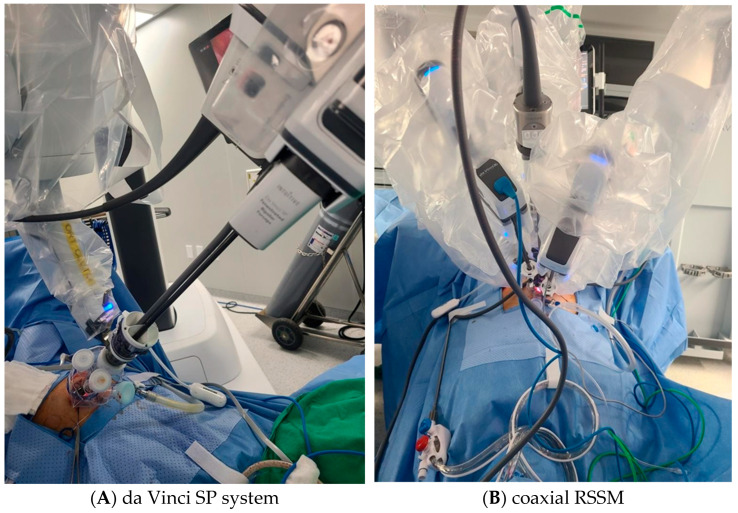
Port configuration for da Vinci SP system (**A**) and coaxial RSSM using da Vinci Xi system (**B**). The SP system uses a single multichannel port through which the camera and instruments are introduced. The coaxial RSSM technique uses separate rigid robotic cannulas aligned coaxially through a single umbilical incision, allowing the use of standard 8 mm instruments.

Each of the three 8 mm robotic cannulas, comprising a regular-length 10 cm cannula for the camera port, another 10 cm cannula for one instrument, and a long 15 cm cannula for the other instrument, was placed into the channels of the glove port. The patient-side cart was docked onto the three cannulas: arm 1.3 to the 15 cm long cannula and arm 2 to the camera-port cannula. To reduce tool interference, a 30-degree upward-view camera scope was utilized. Conventional 8 mm instruments such as a robotic tenaculum or fenestrated bipolar forceps, and wristed monopolar, curved scissors, or mega-needle driver were selectively attached to arms 1 and 3 based on procedural requirements (Figure 1B).

The uterine defect was closed in multiple layers using a continuous technique with either 2-0 V-Loc™ (Covidien, Dublin, Ireland) or 2-0 Monofix^®^ (Samyang, Seoul, Republic of Korea). The abdominal wall was subsequently reapproximated by suturing the peritoneum, fascial layer, and subcutaneous tissue with 1-0 Vicryl^®^ (Ethicon, Raritan, NJ, USA), and the skin was closed using 3-0 Vicryl^®^.

#### 2.3.2. Da Vinci SP

The da Vinci SP robotic platform was docked in the midline, and a cannula featuring four channels inserted into the SP entry system. Within this setup, a 12 mm SP camera and three 6 mm robotic instruments were each inserted into separate channels of the cannula. Articulating instruments in the da Vinci SP system were arranged with monopolar curved scissors at 3 o’clock position, fenestrated bipolar forceps at 9 o’clock, and a needle driver at 6 o’clock within the robotic port layout (Figure 1A). The arms of the monopolar curved scissors and needle driver were interchanged for suturing procedures. The surgical assistant used the remaining trocar of the SP entry system for tasks such as endoscopic suction, placement of suture materials in the pelvic cavity, providing counter-traction with a laparoscopic grasper. Following myoma removal, the uterine wall, including the myometrium and serosa, was repaired in two or three layers using continuous barbed sutures as previously described. The retrieved myomas were enclosed in an endopouch, manually morcellated using a scalpel, and extracted through the umbilical incision. The peritoneum, fascia, and subcutaneous layers were closed as described above.

### 2.4. Statistical Analysis

Continuous variables are presented as mean ± standard deviation. Statistical comparisons were performed using the Mann–Whitney U test for continuous variables, the χ^2^ test for categorical variables, and Fisher’s exact test for non-parametric statistics. To minimize selection bias of surgical outcomes, 1:1 propensity score matching (PSM) was conducted based on total myoma number, largest myoma size, and FIGO classification type between the coaxial RSSM and da Vinci SP groups. All statistical analyses were conducted using SPSS version 24.0 (IBM Inc., Armonk, NY, USA) with statistical significance set at *p* value < 0.05.

## 3. Results

### 3.1. A One-to-One Surgeon Comparative Analysis (Single Surgeon per Institution)

In total, 45 women received coaxial RSSM, while 108 women underwent surgery with the da Vinci SP system. The initial clinical and surgical profiles of the study participants are presented in Table 1. Compared to the coaxial RSSM group, the da Vinci SP group had a larger largest myoma (8.09 ± 2.43 vs. 6.88 ± 1.67 cm, *p* = 0.001), a greater number of myomas (2.73 ± 2.42 vs. 1.89 ± 1.28 cm, *p* = 0.029), and heavier tumor weight (197.76 ± 167.84 vs. 142.4. ± 110.4, *p* = 0.044). Additionally, the da Vinci SP group had a higher BMI (22.90 ± 4.33 vs. 21.03 ± 2.31, *p* = 0.007). No statistically significant differences were identified between the groups in terms of pelvic adhesion, concomitant surgery, or myoma location.

Table 2 presents the initial clinical and surgical profiles after PSM analysis based on the total number of myomas, the largest myoma, and the type of FIGO classification. Following PSM, no statistically meaningful differences were noted between the two groups regarding the size of the largest myoma, the number of myomas, or the tumor weight.

The overall data indicate that coaxial RSSM had a shorter operative time (91.22 ± 18.25 vs. 148.69 ± 45.62 min, *p* < 0.001), lower estimated blood loss (102.33 ± 61.01 vs. 203.98 ± 163.15 mL, *p* < 0.001), and a smaller Hb decrement (1.69 ± 0.93 vs. 2.84 ± 1.29 mL, *p* < 0.001) compared to da Vinci SP (Table 3). However, the da Vinci SP group had a shorter hospital stay (2.10 ± 0.30 vs. 4.27 ± 0.81, *p* < 0.001) relative to coaxial RSSM. The transfusion rates were statistically similar between the two groups (0, 0% vs. 5, 4.6%, *p* = 0.243).

Following PSM analysis (Table 3), coaxial RSSM continued to demonstrate a shorter operative time (90.57 ± 17.92 vs. 143.05 ± 49.35 min, *p* < 0.001), lower estimated blood loss (102.39 ± 61.72 vs. 153.18 ± 102.27 mL, *p* = 0.006), and a smaller Hb decrement (1.72 ± 0.93 vs. 2.33 ± 0.95 mL, *p* = 0.003) compared to da Vinci SP. However, da Vinci SP maintained a shorter hospital stay (4.27 ± 0.82 vs. 2.07 ± 0.26, *p* < 0.001) relative to coaxial RSSM. There were no significant differences in the transfusion rates between the two groups (0, 0% vs. 2, 4.5%, *p* = 0.494). Postoperative complications including ileus, fever lasting more than three days, and surgical site dehiscence did not differ significantly between the two groups, both before and after PSM.

### 3.2. Four-to-One Surgeon Comparative Analysis (Multiple Surgeons at CHA vs. Single Surgeon at Asan)

A total of 81 women underwent coaxial RSSM by four surgeons at CHA Gangnam Medical Center, while 108 women underwent da Vinci SP by one surgeon at Asan Medical Center.

Table 4 shows the baseline characteristics from the four-to-one surgeon comparative analysis. Similar to the one-to-one surgeon comparison, PSM analysis was conducted based on myoma count, the diameter of the largest one, and the type of FIGO classification. After PSM, there were no differences in characteristics between the two groups except for parity (Table 5). After PSM analysis (Table 6), coaxial RSSM demonstrated a shorter operative time (94.34 ± 21.58 vs. 136.29 ± 39.19 min, *p* < 0.001), lower estimated blood loss (128.46 ± 89.52 vs. 188.97 ± 137.95 mL, *p* = 0.003), and a smaller Hb decrement (1.73 ± 0.83 vs. 2.76 ± 1.31 mL, *p* < 0.001) compared to da Vinci SP. However, the da Vinci SP resulted in shortened duration of hospital stay (4.07 ± 0.76 vs. 2.06 ± 0.24, *p* < 0.001) compared to coaxial RSSM. The transfusion rates showed no significant differences between the two groups (1, 1.5% vs. 2, 2.9%, *p* > 0.999). Postoperative complications including ileus, fever persisting for over 72 h, and incisional disruption did not differ between the two groups in both the unmatched and propensity score-matched cohorts. 

## 4. Discussion

The main finding of this study was that coaxial RSSM has a statistically significantly shorter operative time, lower estimated blood loss, smaller hemoglobin decrement, and lower transfusion rates compared to the da Vinci SP group. However, the hospital stay was significantly shorter in the SP group than in the coaxial RSSM group.

The favorable results in the coaxial RSSM group are likely due to the ability to use a conventional robot instrument. In the coaxial group, the use of an 8 mm tenaculum for traction on the myomas likely led to greater strength and reduced enucleation times. This contributed to shorter operative times and less blood loss, leading to fewer transfusions and a smaller decrease in Hb levels measured the day after surgery. Additionally, another hypothesis suggests that the da Vinci SP system, with its instruments featuring two joints for improved articulation, might offer less effective traction for fibroids compared to coaxial procedures that use straight instruments, which inherently provide more power. Straight instruments, even when of the same type, generally offer more force.

Despite achieving favorable outcomes in operative time, estimated blood loss, Hb decrement, and transfusion rates in the coaxial RSSM group, the da Vinci SP group demonstrated a shorter hospital stay. This difference in hospital duration could be attributed to variations in the postoperative recovery period. Differences in each hospital’s critical pathway protocols may play a significant role. Specifically, CHA Gangnam Medical Center set the hospital stay for 4 nights and 5 days, while Asan Hospital set it for 2 nights and 3 days, indicating a difference in duration.

The difference in surgical outcomes between the two centers could be attributed to variations in surgical expertise. Surgeons with extensive experience in robotic surgery, having performed over 1000 procedures (Pf. Seong), are likely to achieve better surgical outcomes due to their proficiency. In contrast, those with intermediate experience, having conducted approximately 300–500 robotic surgeries, were involved in the procedures analyzed in Table 4, Table 5 and Table 6. This additional analysis demonstrates that the differences observed in surgical outcomes are attributable to the surgical techniques rather than the proficiency of individual surgeons. While a more granular analysis such as surgeon-specific multivariate modeling could provide additional insight, it was not feasible due to the limited number of cases per individual surgeon. Nonetheless, by incorporating data from multiple surgeons with varying experience levels, our study design helps mitigate potential operator-related bias and strengthens the generalizability of the findings.

To the best of our knowledge, this is the first cohort-based analysis evaluating and comparing surgical outcomes between coaxial RSSM and the da Vinci SP platform. To enhance the reliability of the results in comparing the surgical outcomes of coaxial RSSM and da Vinci SP, we conducted both a 1:1 surgeon comparison and an analysis of outcomes performed by multiple surgeons.

This study had a few limitations. The primary one is the retrospective nature of the analysis, along with the relatively small sample size. To address potential bias introduced by the retrospective design, we employed a propensity score matching (PSM) analysis. However, due to the limited sample size, it was not feasible to include all baseline variables in the PSM model. Although BMI and parity showed statistically significant differences between groups before matching, they were excluded from the matching process because including all five covariates—BMI, parity, total number of myomas, largest myoma size, and tumor weight—led to a substantial reduction in matched pairs and statistical power. Instead, we prioritized variables more directly associated with surgical complexity, namely the number of myomas, largest myoma size, and FIGO classification. While the exclusion of BMI and parity may have introduced residual confounding, previous studies suggest that their influence on short-term surgical outcomes in myomectomy is relatively limited. Another limitation is the lack of a detailed breakdown of operative time. Specific components such as the docking time, myoma enucleation time, and suturing time were not separately measured, which limits our ability to precisely assess procedural differences between coaxial RSSM and the da Vinci SP system.

In conclusion, this study provides a comparative analysis of the surgical outcomes between coaxial RSSM and the da Vinci SP systems. The da Vinci SP system demonstrated a notable advantage in reducing hospital stay, whereas coaxial RSSM was associated with a shorter operative time, lower intra-operative blood loss, and a reduced hemoglobin decrement postoperatively. These findings present coaxial RSSM as a potentially efficient alternative for institutions utilizing the da Vinci Xi system, particularly considering the discontinuation of SP instruments for SP surgeries. To further substantiate these advantages and ensure a comprehensive evaluation, prospective studies with larger sample sizes are essential. This research highlights the potential for optimized surgical outcomes, informing future surgical practices and technology development.

## Figures and Tables

**Table 1:** Baseline characteristics of myomectomy patients (one-to-one surgeon comparison).

Characteristics	C-RSSM (*n* = 45)	SP (*n* = 108)	*p*
Age, years	36.42 ± 6.22	35.82 ± 5.14	0.571
BMI, kg/m^2^	21.03 ± 2.31	22.90 ± 4.33	0.007
Parity	0 (0–2)	0 (0–2)	0.002
Previous abdominal surgery			0.28
No	37 (82.2)	97 (89.8)	
Yes	8 (17.8)	11 (10.2)	
Peritoneal adhesion			0.284
No	44 (97.8)	100 (92.6)	
Yes	1 (2.2)	8 (7.4)	
Concurrent surgery			0.849
No	40 (88.9)	97 (89.8)	
Ovarian cystectomy	4 (8.9)	9 (8.3)	
USO	0 (0)	0 (0)	
Focal adenomyomectomy	1 (2.2)	2 (1.9)	
Total myoma, *n*	1.89 ± 1.28	2.73 ± 2.42	0.029
Largest myoma Size, cm	6.88 ± 1.67	8.09 ± 2.43	0.001
Location			0.655
Anterior	22 (48.9)	47 (43.5)	
Posterior	16 (35.6)	40 (37.0)	
Fundal	1 (2.2)	10 (9.3)	
Anterior fundal	5 (11.1)	6 (5.6)	
Posterior fundal	2 (2.2)	5 (4.6)	
Type (FIGO classification)			0.116
Submucosal (type 1–2)	4 (8.9)	4 (3.7)	
Intramural (type 3–5)	34 (75.6)	92 (85.2)	
Subserosal (type 6–8)	7 (15.6)	12 (11.1)	
Tumor weight, g	142.40 ± 110.24	197.76 ± 167.84	0.044

Note: Values are presented as number (%), median (range), or mean ± standard deviation. Abbreviations: BMI, body mass index; FIGO, International Federation of Gynecology and Obstetrics; SP, da Vinci SP robotic system; C-RSSM, coaxial robotic single-site myomectomy; RSSM, robotic single-site myomectomy.

**Table 2:** Baseline characteristics of myomectomy patients (PSM, one-to-one surgeon comparison).

Characteristics	C-RSSM (*n* = 44)	SP (*n* = 44)	*p*
Age, years	36.34 ± 6.27	35.59 ± 4.84	0.532
BMI, kg/m^2^	21.06 ± 2.32	22.75 ± 4.80	0.038
Parity	0 (0–2)	0 (0–2)	0.021
Previous abdominal surgery			0.196
No	36 (81.8)	41 (93.2)	
Yes	8 (18.2)	3 (6.8)	
Peritoneal adhesion			0.196
No	36 (81.8)	41 (93.2)	
Yes	8 (18.2)	3 (6.8)	
Concurrent surgery			>0.999
No	39 (88.6)	39 (88.6)	
Ovarian cystectomy	4 (9.1)	4 (9.1)	
USO	0 (0)	0 (0)	
Focal adenomyomectomy	1 (2.3)	1 (2.3)	
Total myoma, *n*	1.89 ± 1.30	1.95 ± 1.56	0.824
Largest myoma Size, cm	6.92 ± 1.68	7.25 ± 1.99	0.406
Location			0.686
Anterior	21 (47.7)	17 (38.6)	
Posterior	16 (36.4)	19 (43.2)	
Fundal	1 (2.3)	4 (9.1)	
Anterior fundal	5 (11.4)	2 (4.5)	
Posterior fundal	1 (2.3)	2 (4.5)	
Type (FIGO classification)			0.484
Submucosal (type 1–2)	4 (9.1)	3 (6.8)	
Intramural (type 3–5)	33 (75.0)	36 (81.8)	
Subserosal (type 6–8)	7(15.9)	5 (11.4)	
Tumor weight, g	144.73 ± 110.39	156.52 ± 143.49	0.667

Note: Values are presented as number (%), median (range), or mean ± standard deviations. Abbreviations: BMI, body mass index; USO, unilateral salpingo-oophorectomy; FIGO, International Federation of Gynecology and Obstetrics; SP, da Vinci SP robotic system; C-RSSM, coaxial robotic single-site myomectomy; RSSM, robotic single-site myomectomy.

**Table 3:** Surgical outcomes and morbidity (one-to-one surgeon comparison).

Characteristics	Total Data			In PSM Data		
	C-RSSM (*n* = 45)	SP (*n* = 108)	*p*	C-RSSM (*n* = 44)	SP (*n* = 44)	*p*
Operative time, mins	91.22 ± 18.25	148.69 ± 45.62	<0.001	90.57 ± 17.92	143.05 ± 49.35	<0.001
EBL, mL	102.33 ± 61.01	203.98 ± 163.15	<0.001	102.39 ± 61.72	153.18 ± 102.27	0.006
Hemoglobin decrement, g/dL	(t)1.69 ± 0.93 1.69 ± 0.93 (*n* = 45)	(t)2.85 ± 1.30 2.84 ± 1.29 (*n* = 103)	<0.001 <0.001	(t)1.72 ± 0.93 1.72 ± 0.93 (*n* = 44)	(t)2.30 ± 0.95 2.33 ± 0.95 (*n* = 42)	0.005 0.003
Transfusion			0.243			0.494
No	79 (98.8)	103 (95.4)		44 (100)	42 (95.5)	
Yes	0 (0)	5 (4.6)		0 (0.0)	2 (4.5)	
Hospital stay, days	4.27 ± 0.81	2.10 ± 0.30	<0.001	4.27 ± 0.82	2.07 ± 0.26	<0.001
Complications			0.503			0.317
None	44 (97.8)	107 (99.1)		43 (97.7)	44 (100)	
Ileus	1 (2.2)	1 (0.9)		1 (2.3)	0 (0)	
Fever > 3 days	0 (0)	0 (0)		0 (0)	0 (0)	
Wound dehiscence	0 (0)	0 (0)		0 (0)	0 (0)	

Note: Values are presented as number (%), median (range), or mean ± standard deviations. Abbreviations: EBL, estimated blood loss; SP, da Vinci SP robotic system; C-RSSM, coaxial robotic single-site myomectomy; RSSM, robotic single-site myomectomy.

**Table 4:** Baseline characteristics of myomectomy patients (four-to-one surgeon).

Characteristics	C-RSSM (*n* = 81)	SP (*n* = 108)	*p*
Age, years	37.31 ± 6.29	35.82 ± 5.14	0.076
BMI, kg/m^2^	21.73 ± 2.53	22.90 ± 4.33	0.031
Parity	0.41 ± 0.72 (0, 0–2)	0.13 ± 0.46 (0, 0–2)	0.001
Previous abdominal surgery			0.061
No	64 (79.0)	97 (89.8)	
Yes	17 (21.0)	11 (10.2)	
Peritoneal adhesion			0.357
No	78 (96.3)	100 (92.6)	
Yes	3 (3.7)	8 (7.4)	
Concurrent surgery			0.900
No	73 (90.1)	97 (89.8)	
Ovarian cystectomy	6 (7.4)	9 (8.3)	
USO	0 (0)	0 (0)	
Focal adenomyomectomy	2 (2.5)	2 (1.9)	
Total myoma, *n*	2.33 ± 1.90	2.73 ± 2.42	0.207
Largest myoma Size, cm	7.09 ± 1.57	8.09 ± 2.43	0.001
Location			0.454
Anterior	40 (49.4)	47 (43.5)	
Posterior	30 (37.0)	40 (37.0)	
Fundal	1 (1.2)	10 (9.3)	
Anterior fundal	8 (9.9)	6 (5.6)	
Posterior fundal	2 (2.5)	5 (4.6)	
Type (FIGO classification)			0.001
Submucosal (type 1–2)	11 (13.6)	4 (3.7)	
Intramural (type 3–5)	52 (64.2)	92 (85.2)	
Subserosal (type 6–8)	18 (22.2)	12 (11.1)	
Tumor weight, g	148.05 ± 111.71	197.76 ± 167.84	0.016

Note: Values are presented as number (%), median (range), or mean ± standard deviations. Abbreviations: BMI, body mass index; USO, unilateral salpingo-oophorectomy; FIGO, International Federation of Gynecology and Obstetrics; SP, da Vinci SP robot system; C-RSSM, coaxial robotic single-site myomectomy; RSSM, robotic single-site myomectomy.

**Table 5:** Baseline characteristics of myomectomy patients (PSM, four-to-one surgeon).

Characteristics	C-RSSM (*n* = 68)	SP (*n* = 68)	*p*
Age, years	37.04 ± 6.49	35.60 ± 4.87	0.145
BMI, kg/m^2^	21.72 ± 2.67	22.78 ± 4.63	0.102
Parity	0.40 ± 0.69 (0, 0–2)	0.16 ± 0.51 (0, 0–2)	0.026
Previous abdominal surgery			0.074
No	55 (80.9)	63 (92.6)	
Yes	13 (19.1)	5 (7.4)	
Peritoneal adhesion			0.441
No	66 (97.1)	63 (92.6)	
Yes	2 (2.9)	5 (7.4)	
Concurrent surgery			0.610
No	62 (91.2)	51 (89.7)	
Ovarian cystectomy	5 (7.4)	5 (7.4)	
USO	0 (0)	0 (0)	
Focal adenomyomectomy	1 (1.5)	2 (2.9)	
Total myoma, *n*	2.09 ± 1.45	2.15 ± 1.35	0.807
Largest myoma Size, cm	7.32 ± 1.51	7.53 ± 2.28	0.537
Location			0.933
Anterior	32 (47.1)	30 (44.1)	
Posterior	27 (39.7)	27 (39.7)	
Fundal	1 (1.5)	7 (10.3)	
Anterior fundal	6 (8.8)	1 (1.5)	
Posterior fundal	2 (2.9)	3 (4.4)	
Type (FIGO classification)			0.759
Submucosal (type 1–2)	3 (4.4)	4 (5.9)	
Intramural (type 3–5)	50 (73.5)	53 (16.2)	
Subserosal (type 6–8)	15 (22.1)	11 (16.2)	
Tumor weight, g	157.58 ± 115.78	162.38 ± 144.84	0.831

Note: Values are presented as number (%), median (range), or mean ± standard deviations. Abbreviations: BMI, body mass index; USO, unilateral salpingo-oophorectomy; FIGO, International Federation of Gynecology and Obstetrics; SP, da Vinci SP robot system; C-RSSM, coaxial robotic single-site myomectomy; RSSM, robotic single-site myomectomy.

**Table 6:** Surgical outcomes and morbidity (PSM, four-to-one surgeon comparison).

Characteristics	Total Data			In PSM Data		
	C-RSSM (*n* = 81)	SP (*n* = 108)	*p*	C-RSSM (*n* = 68)	SP (*n* = 68)	*p*
Operative time, mins	98.09 ± 27.80	148.69 ± 45.62	<0.001	94.34 ± 21.58	136.29 ± 39.19	<0.001
EBL, mL	142.41 ± 115.99	203.98 ± 163.15	<0.001	128.46 ± 89.52	188.97 ± 137.95	0.003
Hemoglobin decrement, g/dL	(t)1.60 ± 0.91 1.67 ± 0.83 (*n* = 79)	(t)2.85 ± 1.29 2.84 ± 1.29 (*n* = 103)	<0.001 <0.001	(t)1.69 ± 0.88 1.73 ± 0.83 (*n* = 67)	(t)2.78 ± 1.33 2.76 ± 1.31 (*n* = 66)	<0.001 <0.001
Transfusion			0.243			>0.999
No	79 (97.5)	103 (95.4)		67 (98.5)	66 (97.1)	
Yes	2 (2.5)	5 (4.6)		1 (1.5)	2 (2.9)	
Hospital stay, days	4.05 ± 0.72	2.10 ± 0.30	<0.001	4.07 ± 0.76	2.06 ± 0.24	<0.001
Complications			0.838			>0.999
None	80 (98.8)	126 (95.5)		67 (98.5)	67 (98.5)	
Ileus	1 (2.2)	1 (0.9)		1 (1.5)	1 (1.5)	
Fever > 3 days	0 (0)	0 (0)		0 (0)	0 (0)	
Wound dehiscence	0 (0)	0 (0)		0 (0)	0 (0)	

Note: Values are presented as number (%), median (range), or mean ± standard deviations. Abbreviations: EBL, estimated blood loss; SP, da Vinci SP robot system; C-RSSM, coaxial robotic single-site myomectomy

## Data Availability

The data supporting the findings of this study are not publicly available due to privacy and ethical restrictions.
